# Student Enrollment and Teacher Statistics Forecasting Based on Time-Series Analysis

**DOI:** 10.1155/2020/1246920

**Published:** 2020-09-15

**Authors:** Stephanie Yang, Hsueh-Chih Chen, Wen-Ching Chen, Cheng-Hong Yang

**Affiliations:** ^1^Department of Educational Psychology and Counseling, National Taiwan Normal University, Taipei 10610, Taiwan; ^2^Institute for Research Excellence in Learning Sciences, National Taiwan Normal University, Taipei, Taiwan; ^3^Chinese Language and Technology Center, National Taiwan Normal University, Taipei, Taiwan; ^4^MOST AI Biomedical Research Center, Taipei, Taiwan; ^5^Department of Electronic Engineering, National Kaohsiung University of Science and Technology, Kaohsiung 80778, Taiwan; ^6^Department of Biomedical Engineering, Kaohsiung Medical University, Kaohsiung 80708, Taiwan; ^7^Drug Development and Value Creation Research Center, Kaohsiung Medical University, Kaohsiung 80708, Taiwan

## Abstract

Education competitiveness is a key feature of national competitiveness. It is crucial for nations to develop and enhance student and teacher potential to increase national competitiveness. The decreasing population of children has caused a series of social problems in many developed countries, directly affecting education and com.petitiveness in an international environment. In Taiwan, a low birthrate has had a large impact on schools at every level because of a substantial decrease in enrollment and a surplus of teachers. Therefore, close attention must be paid to these trends. In this study, combining a whale optimization algorithm (WOA) and support vector regression (WOASVR) was proposed to determine trends of student and teacher numbers in Taiwan for higher accuracy in time-series forecasting analysis. To select the most suitable support vector kernel parameters, WOA was applied. Data collected from the Ministry of Education datasets of student and teacher numbers between 1991 and 2018 were used to examine the proposed method. Analysis revealed that the numbers of students and teachers decreased annually except in private primary schools. A comparison of the forecasting results obtained from WOASVR and other common models indicated that WOASVR provided the lowest mean absolute percentage error (MAPE) and root mean square error (RMSE) for all analyzed datasets. Forecasting performed using the WOASVR method can provide accurate data for use in developing education policies and responses.

## 1. Introduction

For decades, the near-global decline in fertility has led to considerable socioeconomic changes. The low fertility rate observed in many countries is likely the result of economic, social, cultural, and institutional transformations [1]. Some theories linking broad social changes to fertility decline may be relevant to all countries. Common trends for fertility patterns are also present in many regions. Other theories discuss the situation unique to a particular country. Although the fertility transition has taken place globally, the rate of fertility decline, levels that have been hit, and current fertility rates differ by country. The decline in fertility rates in certain societies is likely to result from an interplay of global phenomena, regional policies, and local forces. Weakening economic and global competitiveness and decreasing birthrates will challenge the competitiveness of a nation by leaving it with a labor shortage.

Although demographic change is considered a constant force, educational institutions are pioneers in each generation's shifting composition. Demographers have predicted that schools will change significantly, which will have an impact on the general public as well. The number of students attending school in Taiwan has decreased, which inevitably has an effect on teacher training programs. Given the difference between the teachers' supply and demand, the cultivation of teachers is ultimately affected. The out-of-balance teaching market in Taiwan is a problem that demands attention from the government, educational administrators, and educators, all of whom are responsible for implementing new strategies to rebalance the teaching market.

Current education sustainability heavily depends on the strategic and budgetary planning of each institution, particularly for student enrollment and teacher recruitment. For instance, colleges and universities must achieve enrollment goals annually to achieve the institutional mission and maintain economic vitality. Teacher statistics also require considerable attention to understand the retirement wave and status of the current teaching market. With a view to proposing the most suitable strategies, administrators turn to enrollment professionals to forecast prospective numbers of students and teachers, built on their area of interest. Using this information will assist administrations in accurately distributing resources and constructing future decisions. The forecast presented in this paper can be adapted to project new enrollment or recruitment goals for any given term, regardless of institution type.

Time-series analysis can be used for the trend analysis of time-series data. Time-series data refer to data that are arranged according to a series of time periods or intervals. Time-series analysis involves testing linear or nonlinear relationships among dependent variables. Many linear and nonlinear approaches have been proposed for forecasting in various time-series studies [2, 3]. Linear approaches include exponential smoothing (ETS) [4] and regression-based models, such as autoregressive integrated moving average (ARIMA) [5] and Trigonometric Seasonal Box–Cox Transformation with ARMA residuals Trend and Seasonal Components (TBATS) [6]. The ETS method, which was proposed in 1963 by Brown [4], is a data averaging approach that considers three factors: the error, trend, and season. The maximum likelihood estimation is used in ETS to optimize initial values and parameters, and the optimal ETS model is selected. Moreover, the weight of ETS data decays exponentially. The ARIMA model, which is a well-known time-series prediction method, was proposed by Box and Jenkins [5]. In the ARIMA method, several fragments formed after a time series that has passed are used as input. Moreover, regression analysis is performed to establish a mathematical prediction model, which is often used for the prediction of short-term economic trends. TBATS, which was proposed by Livera in 2011 [6], is a novel method that integrates trigonometric seasonality, Box–Cox transformation, ARIMA error trends, and seasonal components. The TBATS model is an extension of ETS. The TBATS model can forecast whether seasonal data exist and can analyze the data. These methods, which are heavily dependent on linear assumptions, involve using historical datasets to forecast future flow through a univariate or multivariate mathematical function. However, these models are theoretically linear and can be hindered by their weak nonlinear fitting capabilities. The linear assumption makes it difficult for the aforementioned models to process complex nonlinear problems and obtain ideal prediction results.

The spatiotemporal pattern mining method is applied to track the sequence of frequently occurring events in spatiotemporal datasets. Many spatiotemporal pattern-based forecasting and detection methods have been proposed to the prediction accuracy of the sequence of frequently occurring events. For instance, Dubey et al. used a fast and accurate wide-area backup protection scheme for transmission lines based on the synchronized phasor measurement [7]. Cui et al. integrated the spatiotemporal model of system measurement into a flexible Bayes classifier for network attack detection [8]. Sun et al. also used an optimized temporal-spatial scheduling strategy, in the presence of distributed generators, to schedule appropriate charging requirements of plug-in electric vehicles [9]. Furthermore, Cui et al. used the generalized graph Laplacian matrix to visualize the spatiotemporal relationship of the distributed layer phasor measurement unit data [10]. Reinforcement learning (RL) is a powerful technique in machine learning that helps generalization because it enables the design of model-free methods. In recent years, various studies have been conducted using RL. Oh and Wang proposed an RL-based energy storage systems operation strategy which was used to investigate the wind power generation forecast uncertainty [11]. Chen et al. proposed an offline predetermination-online-practice mode and embodied it as model-free control based on RL [12]. Another type of powerful machine learning technique is extreme learning (EL). The calculation is based on a single hidden layer feedforward neural network, which calculates the random weight between the input layer and the hidden layer. By using the EL method and error correction technique, Li et al. proposed a combined statistical method for wind power forecasting [13]. Nonlinear approaches such as radial basis function (RBF) neural networks, multilayer perceptron (MLP) neural networks [14, 15], SVR [16], bagging predictors [17], and regression-based trees [18] have attracted considerable research interest. These approaches have demonstrated sufficient nonlinear fitting capabilities for forecasting demand. The SVM method, which was proposed by Valdimir and Vapnik in 1995 [2], maps all data points into a high-dimensional space and then generates a hyperplane to maximize the boundary between two classes and separate them. SVR was proposed by Vapnik et al. in 1997 [19]. Compared with SVM [3], SVR comprises loss functions, penalty factors, and other elements for improving the robustness of the model. In the SVR method, the total distance from each point to a hyperplane is calculated after mapping data points to a high-dimensional hyperplane. The hyperplane with the smallest total distance is the best solution. SVR has been used successfully to solve various problems in numerous fields, such as medicine [20], and has been proven to be a superior prediction model for time-series analysis [21] and regression analysis [22]. Research based on ANNs and SVR, which have promising nonlinear adaptability, has widened the application of nonlinear methods. Cang et al. proposed a nonlinear combination method that involves the use of MLP neural networks to map the nonlinear relationship between inputs and outputs [23]. Oliveira suggested that SVR can be used to estimate the software project effort. The findings of Oliveira indicate that the performance of SVR is superior to that of RBF neural networks [24]. However, the use of inappropriate parameter settings influences the implementation of ANN and SVR methods. Studies have indicated that the accuracy of the aforementioned methods strongly depends on the values of their hyperparameters. Therefore, thorough guidelines must be developed [25, 26].

Hyperparameter optimization methods have attracted considerable research attention and have been applied in various areas. Many machine learning algorithms, such as the genetic algorithm (GA) [27], particle swarm optimization (PSO) [28], and differential evolution (DE) algorithm [29], have been proposed for optimizing SVM hyperparameters. Luo introduced a novel artificial intelligence approach for predicting the vertical load capacity of driven piles in cohesionless soils through SVR optimized by the GA [30]. Huang et al. combined the PSO algorithm with a backpropagation neural network to establish a demand estimation model [31]. Furthermore, Hasanipanah et al. proposed a new hybrid PSO-SVR model for predicting air overpressure caused by mine blasting [32]. Kuo and Li presented a three-stage method that integrates wavelet transforms, firefly algorithm-based *K*-means algorithms, and firefly algorithm-based SVR for forecasting Taiwanese exports [33]. Seyedpoor proposed a combination of SVR and the DE algorithm for identifying damage in moment frame connections [34]. Support vector regression (SVR) was proposed by Vapnik et al. in 1997 [19]. The SVR algorithm includes the insensitive loss and penalty factor functions; thus, it has higher robustness than the support vector machine (SVM) algorithm [2, 3]. SVR has been proven to be a superior forecasting model for time series [21] and regression analysis [22]. It has three hyperparameters: the regularization parameter (*C*), bandwidth of the kernel function (*σ*), and tube size of the *ε*-insensitive loss function (*ε*). These hyperparameters considerably affect the accuracy of SVR forecasting. However, automatic adjustment of the aforementioned three hyperparameters in SVR remains a prominent challenge for improving the accuracy of SVR forecasting. The whale optimization algorithm (WOA) exhibits a superior performance to other well-known heuristic methods in solving global optimization problems [35] because it can strike a balance between exploitation and exploration during iterations [36]. The WOA can effectively avoid the problem of local optima and maintain rapid convergence. The WOA can be used with an optimization algorithm to avoid the selection of unsuitable hyperparameters, which can result in overfitting or underfitting [37]. Furthermore, the WOA has the advantages of global optimization capabilities, few control hyperparameters, and easy implementation. The WOA has been successfully applied in various optimization problems, such as photovoltaic cell parameter estimation [38], wind speed prediction [39], and energy-related carbon dioxide emission prediction [40]. In this study, we propose the WOASVR algorithm, which is a combination of the WOA and SVR algorithm, for the high-accuracy time-series forecasting analysis of student enrollment and teacher statistics in Taiwan. The WOA was used to obtain suitable hyperparameters for SVR. Experimental results indicated that the WOA outperformed the GRID and PSO algorithms in terms of reducing regression errors in SVR parameter estimation.

## 2. Related Works

Fertility rates are decreasing worldwide. The world's total fertility rate dropped from 5.0 children per woman in 1960 to 2.5 children per woman in 2014 [41]. In the early 1970s, about 43% of the world's population lived in high-fertility countries where women on average had five or more children over their reproductive years and about 18% lived in countries with fertility rates below the replacement level (i.e., 2.1 children per woman) [42]. At present, approximately 46% of the world's population live in countries with subreplacement fertility and only 8% live in high-fertility countries [42]. As a country enters into demographic transition, its fertility may decline. What was probably unexpected was the enormous population affected by the decline in this short period of time [43–45], which is as Caldwell [46] described “unpredicted and unprecedented.”

If the fertility rate continues to remain at such an ultralow level, high-income Asian economies will face serious challenges resulting from depopulation and rapid aging. The shrinking labor force and increasing economic burden of supporting elderly people will pose serious threats to their socioeconomic development and sustainability. At present, governments in Asian societies with ultralow fertility recognize the need to raise fertility, but “exactly what should be done remains elusive” [47, 48]. Formulation of effective population and fertility policies entails a good understanding of the commonalities and uniqueness of the situation in East and Southeast Asia, in comparison with Western countries.

McDonald [49] has pointed out that low fertility rates in Asian societies are related to (1) rising economic risk and insecurity (particularly after the 1997 Asian financial crisis); (2) the difference in gender equality at home and at work; and (3) the lack of support from governments, employers, and society for family needs of young adults [49]. In Asian countries, marriage is often seen as a package of child-rearing and bearing, caring for seniors, and other family obligations, which puts a much heavier burden on women than on men [45]. Furthermore, these societies place high societal expectations on children's education and career achievements, which places great pressure on parents, particularly mothers [50].

As the country with the lowest birthrate [51], the birthrate of the Taiwanese has become the primary problem of population change. Taking a long view, the low birthrate will not be a short-term problem for Taiwan, but a wave of shocks to the future. In education, primary, secondary, and tertiary education institutions are facing challenges that they have not seen in the past. The education problems faced by the younger generation include a decrease in the number of students enrolled and a surplus of teachers [52]. As a result of the low birthrate, primary schools have reported that the wave of decline has affected heavily [48], and with the instability of the retirement system resulting from previously announced adjusted pension policies, the older group of school teachers retired several years ago [53]. Therefore, the faculty in primary schools represents a younger population, and more teachers are available than are required; this has resulted in a wide range of “stray” teachers, and those with a teaching license are yet unable to obtain a formal position due to a lack of vacancies [52]. Without new vacancies opening in the near future and with birthrates continuing to decline, the problem of surplus education graduates and excess of existing teachers demands immediate attention from authorities. In the aspect of the reduction of student enrollment, schools must face the phenomenon of reducing the number of classes year by year, which also implies that school funding cuts are likely to occur [54]. This is happening not only in urban schools; schools in rural areas are battling the crisis more intensively. Many more agricultural counties, such as Kaohsiung, Pingtung, and Tainan counties, have faced a tendency of combining multiple smaller schools as a type of policy adjustment [55]. With the survival of many schools at risk, the politics of education reform will without doubt become significantly more difficult for the Taiwan government.

## 3. Proposed Framework

### 3.1. SVR

SVM is a machine learning method based on statistical learning theory (Vapnik–Chervonenkis theory) and structural risk minimization [56]. The concept aims to find the support vector in the data space to distinguish two different categories to construct a hyperplane in the high-dimensional feature space. This hyperplane can maximize the boundary distance between the two categories and distinguish the two categories of data correctly to obtain high classification accuracy.

In 1997, the introduction of Vapnik's insensitive loss function *ε* [57] was extended to solve nonlinear regression estimation and time-series prediction. The basic idea is to give a set of data (*x*_*i*_, *y*_*i*_), ..., (*x*_*n*_, *y*_*n*_), where *x*_*i*_ ∈ R^*d*^ and *y*_*i*_ ∈ R, *x*_*i*_ is the input vector, *y*_*i*_ is the target value, *i* = 1, 2,…, *n*, where *n* is the sample size of the training data, the *x* is transformed into a high-dimensional feature space *F* mapping through a nonlinear mapping *ϕ*(*x*), and linear regression is performed in the high-dimensional feature space. The SVR linear function is as follows:

(1) $$\begin{array}{c} f\left(x\right)=w\cdot \phi \left(x\right)+b, \end{array}$$

where *w* is a weight vector, which represents the flatness of *f*(*x*) in a high-dimensional space, and *b* is a deviation value;  *ϕ* represents a high-dimensional feature space, which is a nonlinear mapping of the input space *x*. The coefficients of the parameters *w* and *b* can be estimated by minimizing the structural risk. The formula is as follows:

(2) $$\begin{array}{c} R_{\text{reg}}\left(\text{C}\right)=R_{\text{emp}}\left(C\right)+\frac{1}{2}w^{2}\;=\frac{C}{n}\sum_{i=1}^{n}{\left|y_{i}-\;f\left(x\right)\right|}_{\varepsilon }+\frac{1}{2}w^{2}, \end{array}$$

(3) $$\begin{array}{c} {\left|y-f\left(x\right)\right|}_{\varepsilon }=\left\{\begin{array}{cc} 0, & \left|y-f\left(x\right)\right|\leq \varepsilon ,\\ \left|y-f\left(x\right)\right|-\varepsilon , & \text{otherwise,} \end{array}\right. \end{array}$$

where *R*_reg_(*C*) *and* *R*_emp_(*C*) represent the regression error and empirical error, respectively, which are calculated by the *ε*-insensitive loss function as equation (3). (1/2)*w*^2^ is the penalty term; *C* is the penalty constant, which is used to control the degree of error penalty. The penalty term is traded off from the empirical error. To obtain *w* and *b*, a relaxation variable is added to equation (4). The formula is as follows:

(4) $$\begin{array}{c} \begin{array}{cc} \text{minimize} & R_{\text{reg}}\left(w,\xi ,\xi^{*}\right)=C\sum_{i=1}^{n}\left(\xi_{i}+\xi_{i}^{*}\right)+\frac{1}{2}w^{2},\\ \text{subject to} & \left\{\begin{array}{c} -f\left(x_{i}\right)+w\phi \left(x_{i}\right)+b_{i}\leq \varepsilon +\xi_{i}^{*},\\ f\left(x_{i}\right)-w\phi \left(x_{i}\right)-b_{i}\leq \varepsilon +\xi_{i},\\ \xi_{i},\xi_{i}^{*}\geq 0,\;\left(i=1,2,\ldots ,n\right), \end{array}\right. \end{array} \end{array}$$

where *ξ*_*i*_ and *ξ*_*i*_^*∗*^ are imported to measure all training data that fall outside the *ε*-insensitive loss interval. With Lagrange multipliers, satisfy *α*_*i*_ × *α*_*i*_^*∗*^=0, *α*_*i*_ ≥ 0, *α*_*i*_^*∗*^ ≥ 0, and include them into equation (1), as in the following equation:

(5) $$\begin{array}{c} f\left(x\right)=\sum_{i=1}^{n}\left(\alpha_{i}-\alpha_{i}^{*}\right)\left(\phi \left(x_{i}\right)\cdot \phi \left(x\right)\right)+b=\sum_{i=1}^{n}\left(\alpha_{i}-\alpha_{i}^{*}\right)K\left(x_{i},x\right)+b, \end{array}$$

with the Lagrange multiplier brought into equation (4) to obtain the maximal dual equation. The formula is as follows:

(6) $$\begin{array}{c} \begin{array}{cc} \text{maximize} & R\left(\alpha_{i},\alpha_{i}^{*}\right)=\overset{n}{\underset{i=1}{\sum }}y_{i}\left(\alpha_{i}-\alpha_{i}^{*}\right)-\varepsilon \sum_{i=1}^{n}\left(\alpha_{i}+\alpha_{i}^{*}\right)-\frac{1}{2}\sum_{i=1}^{n}\sum_{j=1}^{n}\left(\alpha_{i}-\alpha_{i}^{*}\right)\left(\alpha_{j}-\alpha_{j}^{*}\right)K\left(x_{i},x_{j}\right),\\ \text{subject to} & \left\{\begin{array}{c} \sum_{i=1}^{n}\left(\alpha_{i}-\alpha_{i}^{*}\right)=0,\\ 0\leq \alpha_{i}\leq C,\;\left(i=1,2,\ldots ,\;n\right),\\ 0\leq \alpha_{i}^{*}\leq C,\;\left(i=1,2,\ldots ,\;n\right), \end{array}\right. \end{array} \end{array}$$

where *K*(*x*_*i*_, *x*_*j*_) is defined as the kernel function, and its value is the inner product of two vectors in the feature space *ϕ*(*x*_*i*_) and *ϕ*(*x*_*j*_). The kernel function can avoid complex calculations in high-dimensional spaces. In this study, a Gaussian radial basis kernel function (RBF) is used:

(7) $$\begin{array}{c} K\left(x_{i},x_{j}\right)=\exp\left(\frac{{\left\|x_{i}-x_{j}\right\|}^{2}}{2\sigma^{2}}\right), \end{array}$$

where  *σ* is the bandwidth of the RBF kernel function.

### 3.2. WOA

WOA was proposed by Mirjalili and Lewis [58]. It was inspired by whales' upward spiral bubble-net hunting behavior. In WOA, every humpback whale in the search space is the candidate solution of the optimization problem. The search whale is used to determine the global optimal solution. Given the initial random candidate solution, WOA updates the candidate solution until the end condition is met. A humpback whale randomly swims to search for prey, and spiral bubble-net predation establishes a mathematical model. The WOA has three different behavior patterns: encircling prey, bubble-net attack method, and search for prey. The details of the three behavior patterns are introduced subsequently.

#### 3.2.1. Encircling Prey

The humpback whales identify the prey position and surround the prey. It is assumed that the current position of the best individual whale is the position of target prey or closest to the target prey. Other whales in the population update their positions according to the current position of the prey. The updating function can be formulated by

(8) $$\begin{array}{c} \overset{⟶}{D\;}=\left|\overset{⟶}{C\;}\cdot \overset{⟶}{X^{*}}\left(t\right)-\overset{⟶}{X\;}\left(t\right)\right|, \end{array}$$

(9) $$\begin{array}{c} \overset{⟶}{X\;}\left(t+1\right)=\overset{⟶}{X^{*}}\left(t\right)-\overset{⟶}{A\;}\cdot \overset{⟶}{D}, \end{array}$$

where *t* is the current iterations, $\overset{⟶}{A\;}$ and $\overset{⟶}{C\;}$ are coefficient vectors, $\overset{⟶}{X^{*}}$ is the current best solution position vector, and $\overset{⟶}{X\;}$ is the current solution position vector. The coefficient vectors $\overset{⟶}{A\;}$ and $\overset{⟶}{C\;}$ are as follows:

(10) $$\begin{array}{c} \overset{⟶}{A\;}=2\overset{⟶}{a\;}\cdot \overset{⟶}{r\;}-\overset{⟶}{a\;}, \end{array}$$

(11) $$\begin{array}{c} \overset{⟶}{C\;}=2\cdot \overset{⟶}{r\;}, \end{array}$$

(12) $$\begin{array}{c} \overset{⟶}{a\;}=2-\left(\frac{2t}{\text{Max}\_t}\right), \end{array}$$

where $\overset{⟶}{r\;}$ is a random vector between [0, 1], $\overset{⟶}{a\;}$ is linearly reduced from 2 to 0 during the iteration, and *Max*_*t*_ is the maximum iterations.

#### 3.2.2. Bubble-Net Attacking Method

According to the foraging behavior of humpback whales using a bubble net, two kinds of behavior mechanisms exist: a shrinking encircling mechanism and a spiral updating position. The positions of contraction encirclement and spiral renewal are as follows:

(1) Shrinking encircling mechanism: when $\overset{⟶}{a\;}$ decreases linearly from 2 to 0 during the iteration process, this behavior reduces $\overset{⟶}{A\;}$ in equation (10) by equation (12) to achieve contraction envelopment, in which $\overset{⟶}{A\;}$ is [−a, a] random value within the interval. Therefore, by setting the random value $\overset{⟶}{A\;}$ between [−1, 1], the position of the individual whale group appears at any position between the current position and the current position of the best solution.
(2) Spiral updating position: this stage calculates the distance between the individual whale group and its prey and then creates a spiral model between the individual whale group and its prey position to simulate the spiral swimming behavior of the humpback whale. The model can be formulated by
  (13) $$\begin{array}{c} \overset{⟶}{X\;}\left(t+1\right)=\overset{⟶}{D^{\prime }}\cdot e^{bl}\cdot \cos\left(2\pi l\right)+\overset{⟶}{X^{*}}\left(t\right), \end{array}$$
  (14) $$\begin{array}{c} \overset{⟶}{D^{\prime }}=\left|\overset{⟶}{X^{*}}\left(t\right)-\overset{⟶}{X\;}\left(t\right)\right|, \end{array}$$

where $\overset{⟶}{D^{\prime }}$ is the distance between the current best position of the individual whale and its prey, *b* is a constant defining the spiral shape, and *l* is a random value between [−1, 1].

When humpback whales shrink around prey and move to feed along the spiral shape path at the same time, it is assumed that the probability of two behavior mechanisms selected in the process of updating the individual position of the whale group is 0.5. The position updating can be formulated by

(15) $$\begin{array}{c} \overset{⟶}{X\;}\left(t+1\right)=\left\{\begin{array}{c} \overset{⟶}{X^{*}}\left(t\right)-\overset{⟶}{A\;}\cdot \overset{⟶}{D\;},\\ \overset{⟶}{D^{\prime }}\;\cdot e^{bl}\cdot \cos\left(2\pi l\right)+\overset{⟶}{X^{*}}\left(t\right). \end{array}\right. \end{array}$$

#### 3.2.3. Search for Prey

When |*A*| ≥ 1, in the stage of searching for prey, individuals of the whale group randomly search for prey according to each other's position. $\overset{⟶}{A\;}$ takes a random value; when it is greater than 1 or less than −1, it forces the whale group to deviate from the prey's location to search for other more suitable prey, so that WOA has the best global search ability. The mathematical model is as follows:

(16) $$\begin{array}{c} \overset{⟶}{D\;}=\left|\overset{⟶}{C\;}\cdot \overset{⟶}{X_{\text{rand}}}-\overset{⟶}{X\;}\right|, \end{array}$$

(17) $$\begin{array}{c} \overset{⟶}{X\;}\left(t+1\right)=\overset{⟶}{X_{\text{rand}}}-\overset{⟶}{A\;}\cdot \overset{⟶}{D\;}, \end{array}$$

where $\overset{⟶}{X_{\text{rand}}}$ is a randomly selected position from the current whale group.

### 3.3. Selecting SVR Parameters Using WOA

**Figure 1:**
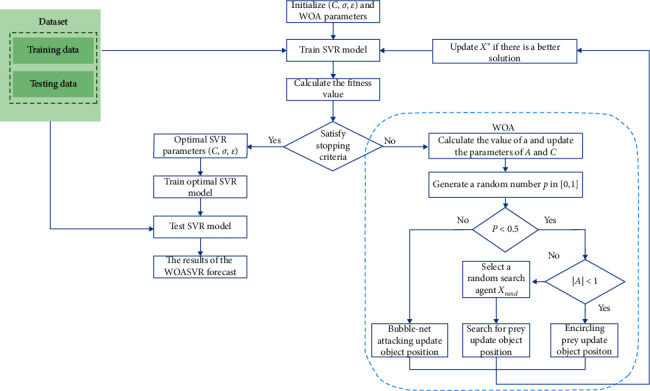
WOASVR flowchart.

The SVR parameters are selected using WOA, as presented in Figure 1 and Algorithm 1. When setting up the SVR model, the parameters can influence the prediction effect. SVR includes three parameters: the *C* penalty constant, the *ε*-insensitive loss function, and the bandwidth of the *σ* kernel function. An improper approach could cause the overfitting or underfitting of parameter selections. In this study, the WOA algorithm was used to select the parameters of the SVR model. Figure 1 illustrates a flowchart of WOASVR. The process is as follows:

  Step 1: the WOA parameters are initialized, and then a whale is randomly generated in the search space. Each whale *i* is represented by *x*_*i*_ = {*C*, *ε*, *σ*}.
  Step 2: to evaluate fitness value, put the three parameters *C*, *ε*, and *σ* into the SVR model to predict the problem, and use *k*-fold cross-validation (CV) during the training phase to avoid overfitting and calculate the validation error value, divide the data randomly into *k* sets, then use one set as testing data and the remaining *k* − 1 sets as training data; repeat until each set has been used as test data. The final prediction result is compared with the actual result. The mean absolute percentage error (MAPE) is used as the adaptation function, and the *k* MAPEs are averaged to obtain the final MAPE_cv_. The value of *k* is set to 4, which is calculated as follows:

(18) $$\begin{array}{c} {\text{MAPE}}_{\text{CV}}=\frac{1}{n}\sum_{i=1}^{n}\left|\frac{y_{i}-f_{i}}{y_{i}}\right|\times 100\%, \end{array}$$

  where *y*_*i*_ is the actual value, *f*_*i*_ is the predicted value, and *n* is the sample size of the test data.
  Step 3: use the fitness function of equation (18) to calculate the fitness value of the individual whale group. The best individual whale group with the best fitness value is saved as *X*^∗^.
  Step 4: if the current number of iterations (*t*) ≤ the maximum number of iterations (Max_*t*_), update *a*, *A*, *C*, *l*, and *p*.
  Step 5: when *p* < 0.5, *A* <  1 uses equation (9) to update the current position of the individual whale group. If *A* ≥ 1, randomly select the individual whale group position *X*_rand_ from the current whale group and use equation (17) to update the current individual whale herd position.
  Step 6: when *p* ≥ 0.5, use equation (13) to update the current position of the individual whale group.
  Step 7: use the fitness function of equation (18) to calculate the fitness value of the individual whale group, and find and save the best individual whale group in the current group (*X*^*∗*^).
  Step 8: determine whether the termination condition is met. If the condition is met, output the adaptive value position *X*^*∗*^ of the individual whale group; otherwise *t* = *t* + 1, repeat steps (4) to (7), and *X*^*∗*^ is the SVR model's optimal parameters.
  Step 9: use the optimal parameters to train the SVR model.
  Step 10: use the best trained SVR model to predict the result.

### 3.4. Performance Criteria

To evaluate the forecast performance of the WOASVR model, two common statistical measures were used in this study, namely, the root mean square error (RMSE) and MAPE, for comparing the deviation of the actual and predicted values. The RMSE and MAPE metrics are expressed in (19) and (20), respectively.

(19) $$\begin{array}{c} \text{RMSE}=\sqrt{\frac{1}{n}\sum_{i=1}^{n}{\left(y_{i}-f_{i}\right)}^{2}}, \end{array}$$

(20) $$\begin{array}{c} \text{MAPE}=\frac{1}{n}\sum_{i=1}^{n}\left|\frac{y_{i}-f_{i}}{y_{i}}\right|\times 100\%, \end{array}$$

where *y*_*i*_ is the actual value, *f*_*i*_ is the predicted value, and *n* is the sample size of the test data.

## 4. Experimental Results

### 4.1. Datasets and Preprocessing

A hybrid model, combination of WOA and SVR (WOASVR), is presented to forecast student enrollment and teacher statistics in Taiwan. To evaluate the proposed approach, we applied it to data on student enrollment and teacher statistics as a case study. From 1999 to 2018, data were collected in a Ministry of Education database, and demographics were categorized for public and private schools [59]. The training data used to train the algorithms consisted of the annual data for 1999–2012. The forecast accuracy was evaluated using the testing data, which consisted of the annual student enrollment and teacher statistics data for 2013–2018.

### 4.2. SVR Parameter Settings

SVR has three hyperparameters: the regularization parameter (*C*), bandwidth of the kernel function (*σ*), and tube size of the *ε*-insensitive loss function (*ε*). These hyperparameters considerably affect the accuracy of SVR forecasting. The traditional SVR model uses a grid search method (GRIDSVR) to determine optimal hyperparameters. GRIDSVR increases the hyperparameters exponentially [60]. Therefore, the search parameters of GRIDSVR were set as follows: *C* = [2^0^, 2^1^, 2^2^,…, 2^22^], *σ* = [2^−10^, 2^−9^, 2^−8^,…, 2^0^], and *ε* = [2^−10^, 2^−9^, 2^−8^,…, 2^0^]. In this study, two popular machine learning algorithms, namely, PSO and WOA, are proposed to optimize SVR hyperparameters. The population size in PSO was set as 50, the acceleration factors *c*_1_ and *c*_2_ were set as 2.0, and the maximum number of iterations was set as 100. The aforementioned hyperparameters were selected in accordance with the study of Bratton and Kennedy [61]. The population size in WOA was set as 20, and the maximum number of iterations was set at 100. The training results obtained from GRIDSVR, PSOSVR, and WOASVR methods with the selected hyperparameters for student and teacher forecasting are presented in Tables 1 and 2, respectively.

### 4.3. Comparison of the ETS, ARIMA, TBATS, GRIDSVR, PSOSVR, and WOASVR

**Figure 2:**
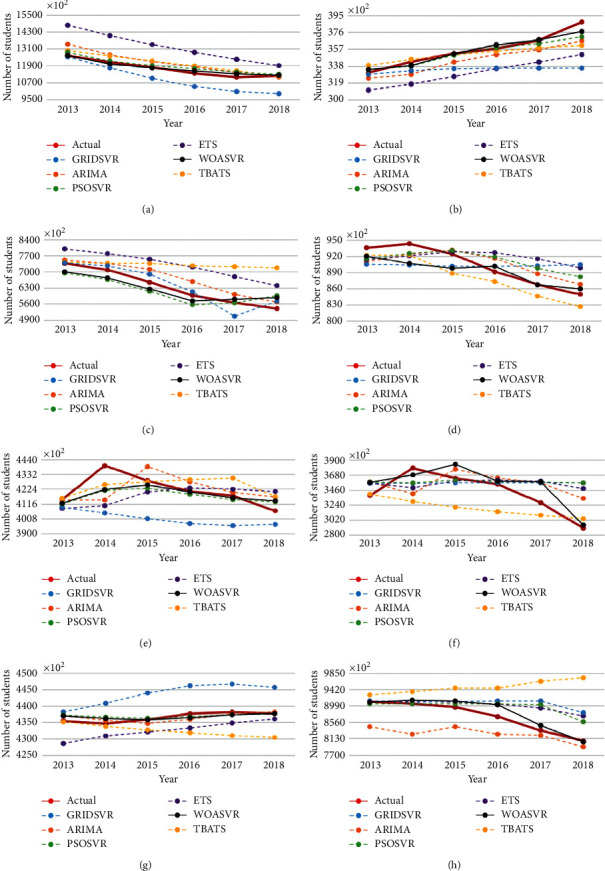
Illustrations of forecast results for student enrollment datasets: (a) primary school-public; (b) primary school-private; (c) middle school-public; (d) middle school-private; (e) high school-public; (f) high school-private; (g) university-public; (h) university-private.

**Figure 3:**
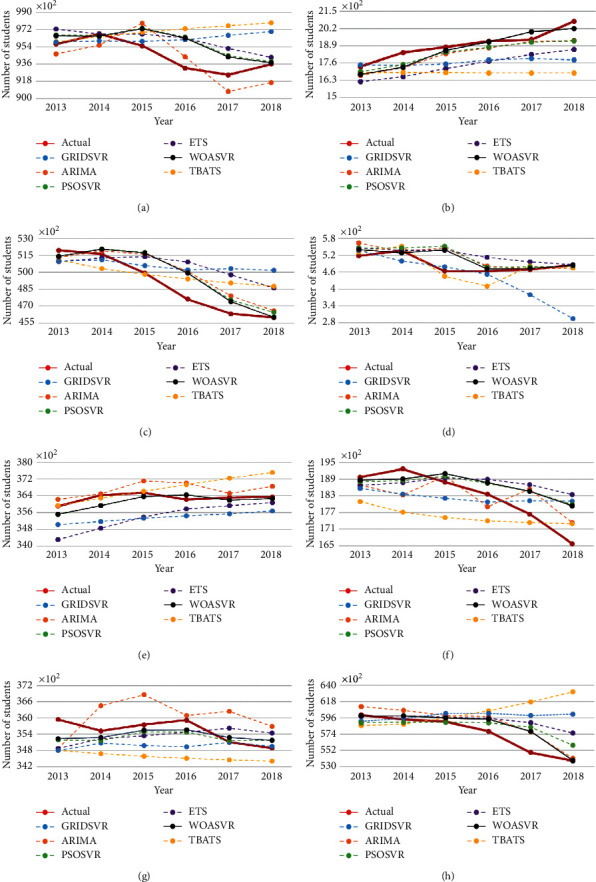
Illustrations of forecast results for teacher datasets: (a) primary school-public; (b) primary school-private; (c) middle school-public; (d) middle school-private; (e) high school-public; (f) high school-private; (g) university-public; (h) university-private.

In this study, a WOASVR model which combined WOA with SVR algorithms was proposed. The WOA method was used to optimize the SVR parameters and enhanced the performance of SVR model. The proposed model was applied to number forecasting of student enrollment and teachers to obtain minimal prediction error. To evaluate the performance of time-series forecast, the proposed model (WOASVR) was compared with an SVR model optimized by PSO or a grid search algorithm and the statistical models included ARIMA [5], ETS [4], and TBATS [6]. Mean absolute percentage error (MAPE) and root mean square error (RMSE) were used as a performance interpretation metric. In a practical time-series forecasting experience, a limited dataset is a frequent drawback for statistical models; it can be quite challenging to achieve the desired results with limited samples. The SVR approach with its strong generalization capability can effectively overcome technical challenges such as minimal datasets and nonlinear, high-dimensional, and local minimum values. Tables 3 and 4 present the average MAPE values of the forecasts obtained using ARIMA, ETS, SVR, PSOSVR, and WOASVR for both datasets (public school and private school). As shown in Table 3, for the public school, the MAPE values of ARIMA, ETS, TBATS, GRIDSVR, PSOSVR, and WOASVR were 3.00, 7.98, 5.65, 5.45, 2.45, and 2.09, respectively. That means the student enrollment forecasting accuracies of the proposed method WOASVR were superior to the other models. For the private school, the student enrollment forecasting accuracies of the proposed method WOASVR (2.11) were also superior to the other models, including ARIMA, ETS, TBATS, GRIDSVR, and PSOSVR, with the MAPE values of 4.50, 5.41, 7.16, 5.53, and 3.62, respectively. For the teacher forecasting accuracies of public school, shown in Table 4, WOASVR (1.39) also revealed a lowest MAPE value compared with ARIMA (1.65), ETS (1.67), TBATS (2.75), GRIDSVR (2.12), and PSOSVR (1.46). The same finding was obtained for the private school, and the MAPE value of WOASVR (2.86) was lower than those of ARIMA (3.77), ETS (5.43), TBATS (11.59), GRIDSVR (7.00), and PSOSVR (3.38). The results suggest that the WOASVR model is the most effective in parameter optimization and is feasible for predicting student enrollment and teacher numbers. Figures 2 and 3 describe the differences between the actual data and the prediction results.

## 5. Discussion

### 5.1. Model Performance

To achieve high efficiency, prediction accuracy, and stability, optimal hyperparameters must be determined for the SVM algorithm. However, the selection of appropriate SVR hyperparameters is a vital challenge. In this study, we proposed the WOASVR algorithm, which is a combination of WOA and SVR algorithms, for the high-accuracy time-series forecasting analysis of the student enrollment and teacher statistics in Taiwan. The algorithm WOA was used to determine suitable hyperparameters for SVR. The predictive power of the WOASVR approach was compared with those of five well-known algorithms: ARIMA, ETS, TBATS, GRIDSVR, and PSOSVR. The datasets of student and teacher numbers between 1991 and 2018 were used to compare the performance of the proposed algorithm with that of the aforementioned five methods. The hyperparameters acquired using the WOASVR model were more accurate than those acquired using the GRIDSVR and PSOVR models; thus, the WOASVR algorithm was more effective on the optimization of SVR hyperparameters than the GRIDSVR and PSOSVR algorithms. The WOA algorithm can provide high local optima avoidance and convergence speed during the course of iteration. Moreover, the WOASVR algorithm achieved higher forecasting accuracy than the other methods. The results indicate that the WOASVR model is a superior method for forecasting student enrollment and teacher statistics.

Only two parameters were considered during the implementation of the WOASVR method: namely, the size of the population in WOA and the maximum number of iterations. A large population size and number of iterations enhanced the searching ability for determining the trends of student and teacher numbers in Taiwan. However, the computational time also increased with the population size and number of iterations. In this study, the WOASVR method required a population size of only 20, whereas the PSOSVR method required a population size of 50. Using the common PSO parameter optimization approaches, the overall performance revealed that WOA achieved a better result and reduced operating costs in fewer iteration. Thus, the parameter-optimizing ability of WOASVR was superior to that of PSOSVR [58]. The high search capability of WOA was due to the population location update mechanism, which is presented in equation (17). Equation (17) requires population to move randomly in the initial steps of the iterative operation. However, in the remainder of the iterative operation, the high development and convergence derived from equation (15) are emphasized. In accordance with equation (15), the population repositions itself around the current best solution or moves in a spiral path to seek the new best solution. Because the aforementioned two stages were conducted separately and each stage only required approximately half number of the iterations for parameter optimization, WOASVR exhibited a better local optimal avoidance and convergence speed than the other methods [58].

### 5.2. Trend Evaluation

In the early 2000s, new education institutions were still opening in Asia to cater to the increasing number of children, particularly the large numbers of babies born in the dragon years 1988 and 2000. Two decades later, many schools are closing. Ministry of education in various countries is left with reluctant decisions regarding shrinking school cohorts and is either closing or merging schools. The considerable decline of birthrates has evidently made an impact on student enrollment, with elementary schools first pushed to the edge. In this study, accurate forecasts from the proposed methodology offer findings that are capable of providing the government, administrators, and educators a picture of future school attendance trends and what these trends mean for the demand for education. Applicable strategies are also provided to accommodate the found trends.

Numerous trends are worth noting. First, as shown in Figures 2(a) and 2(b), the trends for public and private primary student enrollment were in opposite directions. Whereas public school student enrollment is declining, private schools are surprisingly gaining students. This finding can be explained by the emergence of the quantity-quality trade-off theory developed by Becker and associates [62–67]. According to their model, the increasing marginal cost of quality (child output) in terms of quantity (number of children) leads to a trade-off between quantity and quality. Countries with a low fertility rate tend to be wealthier, with higher per capita incomes [67]. Smaller families could invest more in each child, thus improving education, health, and cognitive ability. Compulsory education provided by the Taiwanese state was meant to enable all citizens to receive basic education by providing education based on a standard academic curriculum. Private schools charge parents higher cost tuition than the norm claim to provide students additional assistance in academic training or opportunities to participate in additional extracurricular activities to cultivate additional “talents”. Additional tuition is also often used to provide students better facilities and amenities than those provided to their public counterparts. Our results suggest that with the low fertility rate in Taiwan [51], current Taiwanese parents are willing to invest a relatively large amount of money in their children's education; therefore, they select private schools over public schools for their children's education. The trend of our forecast is consistent with the quantity-quality trade-off theory of Becker et al. [62–67]. Small Taiwanese families, which have strong financial capability, are willing to invest considerable money for their children's education, health, and cognitive skill cultivation.

The numbers of student enrollment and teachers also caught our attention. As shown in Figures 2(c) and 2(d), a quite considerable increase of student enrollment occurred in 2014. Students who were enrolled as freshmen that year were mostly born in 2000, the year of the dragon. There is typically a bump in the number of children born in the dragon year, considered in Chinese culture to be auspicious, which demands that school administrations and governments cater to this projected population. Lastly, another trend (presented in Figure 3) was a major drop in the number of teachers in 2015, possibly the result of retirement waves among public school teachers. In 2013, with the aim to prevent the existing pension system from going bankrupt, new pension policies were introduced by the Taiwanese government, which delayed the official retirement age. Around the same time, in hopes to foster future generations' key competency and the nation's economic competency, a new law named 12-year Curriculum for Basic Education was approved [68]. The law readjusted the Taiwan's education system to align with demographic changes and current problems the island was facing, and many changes were unfamiliar to teachers. Many teachers, under the pressure of facing foreign curriculums and the extended retirement age policy, decided to retire before the law was implemented, hence the considerable drop in the number of teachers. For the many challenges ahead, the following strategies are elaborated for future reference.

### 5.3. Further Strategic Implications

It is inevitable that schools should be repurposed after a declining fertility trend. Schools bolster the local community and support residents' well-being, which contributes to a positive neighborhood [69]; therefore, to repurpose schools, developing community hubs on unused school space is essential [70]. A practical strategy is to place recruitment efforts in searching for new markets. Elder-friendly community models, as defined by the World Health Organization, are ones that promote active aging [71], which is a process that enhances life quality while aging. Engagement in meaningful activities, such as learning, can contribute to good physical and psychological health [72]. With the extra resources and facilities that were once used to accommodate the younger population's education needs, schools can provide the elder population an opportunity to learn as well.

A reform of the higher education system demands attention. Following universal expansion and reform, Taiwan's higher education system has received wide recognition and is considered reputable in Asia. Due to the “410 Demonstration for Education Reform” policy, the number of higher education institutions in Taiwan rapidly increased, from 130 in 1994 to 164 in 2007 [73]. In 2008, the percentage of high school graduates who entered university hit 95%, and it has remained this high since. This policy has then caused a failure of the system to be discriminative, and with the considerable birthrate decline, the future of Taiwan's higher education system is even less positive. Today, facilitating a university elimination mechanism for endangered schools is a necessity for Taiwan, by either shutting down those with poor performance or transforming them into other purposes. A well-thought scanning initiative will decrease wasteful investments and steer effort toward upgrading higher education quality, which is an additional implementation Taiwanese higher education currently needs [74]. Government and school administrators should rethink the concepts and directions of school management and adjust administration strategies and policies in response to the declining birthrate in Taiwan. To increase national competence, accurate forecasting of student enrollment and teacher statistics is critical for ensuring that human resources can be effectively developed in the future.

## 6. Conclusion

Inevitable demographic change is a force all nations must face today. With the phenomenon of a declining birthrate, government education departments, schools, and educators should rethink the concept and direction of school management and adjust administration strategies and policies in response to this social trend. To increase national competence, accurate forecasting of student enrollment and teacher statistics is critical to ensuring that human resources can be effectively developed. In this study, we proposed the WOASVR algorithm which was combined with WOA and SVR for the forecasting of student enrollment and teacher statistics. WOA was used to tune the suitable parameters for SVR. The datasets of student and teacher numbers between 1991 and 2018 were used to evaluate the performance of the proposed algorithm. The predictive power of the WOASVR approach was compared with well-known algorithms in five models: ARIMA, ETS, TBATS, GRIDSVR, and PSOSVR. The parameters acquired using the WOASVR model were more accurate than those acquired using GRIDSVR and PSOVR, which indicates that WOASVR is more effective at optimizing SVR parameters than GRIDSVR or PSOSVR. The results indicate WOA algorithm has the ability to provide high local optima avoidance and convergence speed during the course of iteration. Moreover, WOASVR achieved higher forecasting accuracy than the other methods, indicating that the WOASVR model is a superior method for forecasting student enrollment and teacher statistics.

## Figures and Tables

**Algorithm 1:**
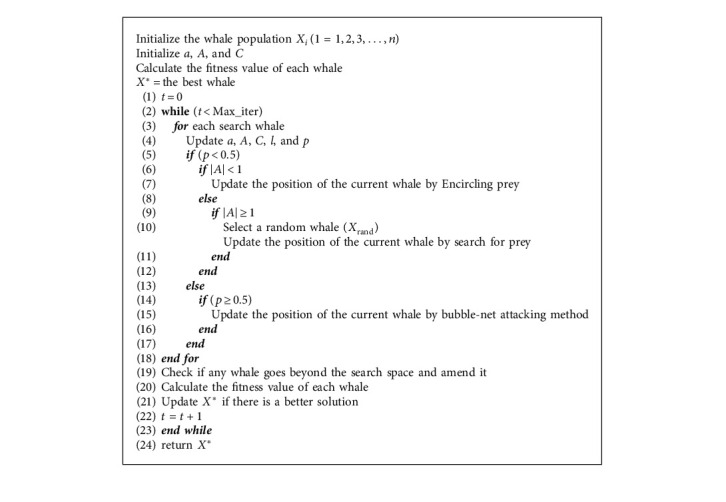
Whale optimization algorithm.

**Table 1:** Training results of GRIDSVR, PSOSVR, and WOASVR under selected parameters (students).

Number of students	*C*			*ε*			*σ*		
	GRIDSVR	PSOSVR	WOASVR	GRIDSVR	PSOVR	WOASVR	GRIDSVR	PSOSVR	WOASVR
Primary school-public	512.00	629793.30	1825063.86	0.0312	0.0261	0.0383	0.0156	0.0047	0.0538
Primary school-private	128.00	93053.61	135594.15	0.1250	0.0227	0.0120	0.0156	0.0254	0.0372
Secondary school-public	256.00	2605425.88	2203505.62	0.0039	0.2257	0.2134	0.0039	0.0157	0.0005
Secondary school-private	2.00	64931.66	55942.95	0.0039	0.0079	0.1100	0.0312	0.0604	0.0345
High school-public	64.00	122065.09	161139.26	0.2500	0.0214	0.0161	0.0039	0.0100	0.0026
High school-private	512.00	1573376.00	66764.71	0.0625	0.6250	0.0952	0.0078	0.0020	0.7245
University-public	512.00	232923.95	67587.11	0.0312	0.1108	0.0878	0.0039	0.0035	0.0059
University-private	512.00	74970.22	24053.28	0.2500	0.1617	0.0227	0.0039	0.0062	0.0157

GRIDSVR, grid search support vector regression; PSOSVR, particle swarm optimization support vector regression; WOASVR, whale optimization algorithm support vector regression; *C*, penalty factor; *ε*, epsilon; *σ*, sigma.

**Table 2:** Training results of GRIDSVR, PSOSVR, and WOASVR under selected parameters (teachers).

Number of teachers	*C*			*ε*			*σ*		
	GRIDSVR	PSOSVR	WOASVR	GRIDSVR	PSOVR	WOASVR	GRIDSVR	PSOSVR	WOASVR
Primary school-public	1024.00	1585.32	4096.00	0.0156	0.2352	0.1458	0.0313	0.0012	0.0111
Primary school-private	512.00	11688.09	388640.22	0.1250	0.0089	0.1053	0.0156	0.0432	0.0056
Secondary school-public	1024.00	39199.21	241867.33	0.0078	0.0273	0.0111	0.0078	0.3034	0.0768
Secondary school-private	16.00	69.35	62.58	1.0000	0.3033	0.2433	0.0313	0.3931	0.1938
High school-public	256.00	97122.75	94308.38	0.0313	0.0079	0.0083	0.0039	0.1453	0.1694
High school-private	1024.00	8192.00	8183.06	0.0156	0.0125	0.0185	0.0039	0.0111	0.0253
University-public	16.00	8447.03	7563.32	0.2500	0.3330	0.1712	0.0156	0.0042	0.8350
University-private	4.00	2072.83	1955.08	1.0000	0.8612	0.6734	0.0039	0.0137	0.0132

GRIDSVR, grid search support vector regression; PSOSVR, particle swarm optimization support vector regression; WOASVR, whale optimization algorithm support vector regression; *C*, penalty factor; *ε*, epsilon; *σ*, sigma.

**Table 3:** Performance comparison of different forecasting models for student enrollment dataset.

Number of students		Criteria	ARIMA	ETS	TBATS	GRIDSVR	PSOSVR	WOASVR
Primary school	Public	MAPE (%)	3.57	12.89	3.11	6.65	1.61	**0.86**
		RMSE	47989.51	159243.65	40320.54	86053.33	21606.28	**12787.62**
	Private	MAPE (%)	3.12	7.16	7.95	6.06	1.46	**1.07**
		RMSE	1243.26	2626.75	3439.16	2761.55	739.57	**528.48**

Secondary school	Public	MAPE (%)	6.03	15.59	16.53	8.74	6.00	**5.57**
		RMSE	40912.00	98097.44	116066.74	74626.56	40615.21	**39029.75**
	Private	MAPE (%)	2.14	3.40	3.72	3.61	2.46	**1.85**
		RMSE	2233.16	3421.01	4051.42	3522.10	2418.49	**2049.95**

High school	Public	MAPE (%)	2.15	2.55	2.03	4.80	1.95	**1.71**
		RMSE	12232.91	14061.67	11797.81	22763.48	12199.88	**11762.71**
	Private	MAPE (%)	7.68	7.51	7.55	7.56	6.75	**4.16**
		RMSE	33472.75	30483.37	32005.37	32036.26	30279.43	**17782.32**

University	Public	MAPE (%)	0.24	0.89	0.91	1.61	0.22	**0.2**
		RMSE	1181.90	4188.08	4933.25	7319.07	1208.03	**1105.6**
	Private	MAPE (%)	5.06	3.56	9.43	4.87	3.80	**1.36**
		RMSE	50997.54	39093.13	95690.97	56050.40	48495.19	**15621.16**

Average	Public	MAPE (%)	3.00	7.98	5.65	5.45	2.45	**2.09**
		RMSE	25579.08	68897.71	43279.59	47690.61	18907.35	**16171.42**
	Private	MAPE (%)	4.50	5.41	7.16	5.53	3.62	**2.11**
		RMSE	21986.68	18906.07	33796.73	23592.58	20483.17	**8995.48**

ARIMA, autoregressive integrated moving average; ETS, exponential smoothing; TBATS, Trigonometric Seasonal Box–Cox Transformation with ARMA residuals Trend and Seasonal Components; GRIDSVR, grid search support vector regression; PSOSVR, particle swarm optimization support vector regression; WOASVR, whale optimization algorithm support vector regression; boldface, the best values in each row.

**Table 4:** Performance comparison of different forecasting models for teacher dataset.

Number of teachers		Criteria	ARIMA	ETS	TBATS	GRIDSVR	PSOSVR	WOASVR
Primary school	Public	MAPE (%)	1.65	1.67	2.75	2.12	1.46	**1.39**
		RMSE	1835.08	1915.49	3259.28	2522.98	1769.12	**1702.23**
	Private	MAPE (%)	3.83	8.15	10.88	6.92	3.44	**2.86**
		RMSE	84.62	159.46	236.42	158.92	77.21	**61.8**

Secondary school	Public	MAPE (%)	2.50	4.27	6.54	4.60	2.25	**2.04**
		RMSE	1427.81	2348.59	3901.30	2665.91	1340.90	**1306.95**
	Private	MAPE (%)	5.57	6.26	13.03	12.42	4.61	**3.93**
		RMSE	39.06	41.06	68.30	87.88	38.59	**32.67**

High school	Public	MAPE (%)	1.13	2.48	1.40	2.55	0.7	**0.69**
		RMSE	474.53	1058.47	675.91	953.11	292.2	**288.08**
	Private	MAPE (%)	3.27	4.10	4.38	3.91	3.09	**3.07**
		RMSE	757.49	906.79	1094.97	815.19	712.99	**702.78**

University	Public	MAPE (%)	2.45	1.54	2.65	1.59	1.31	**1.21**
		RMSE	931.06	600.89	999.42	715.22	527.44	**471.26**
	Private	MAPE (%)	2.42	3.22	18.08	4.76	2.36	**1.58**
		RMSE	1614.25	2379.39	11928.49	3482.68	1773.46	**1376.06**

Average	Public	MAPE (%)	1.93	2.49	3.34	2.72	1.43	**1.33**
		RMSE	1167.12	1480.86	2208.98	1714.31	982.42	**942.13**
	Private	MAPE (%)	3.77	5.43	11.59	7.00	3.38	**2.86**
		RMSE	623.86	871.68	3332.05	1136.17	650.56	**543.33**

ARIMA, autoregressive integrated moving average; ETS, exponential smoothing; TBATS, Trigonometric Seasonal Box–Cox Transformation with ARMA residuals Trend and Seasonal Components; GRIDSVR, grid search support vector regression; PSOSVR, particle swarm optimization support vector regression; WOASVR, whale optimization algorithm support vector regression; boldface, the best values in each row.

## Data Availability

The data supporting this study are openly available from the Ministry of Education, ROC, Taiwan, at https://english.moe.gov.tw/mp-1.html.

## Supplementary Materials

Supplementary Materials The supplementary figures demonstrate (1) the number of students enrolled (Supplementary Figure 1) and full-time equivalent teachers hired (Supplementary Figure 2) in Taiwanese public and private educational institutions; (2) student-teacher ratio in public (Supplementary Figure 3) and private (Supplementary Figure 4) educational institutions; and (3) the trend of student and teacher statistics in public and private educational institutions based on level (Supplementary Figures 5–8).
