# Supplementary material for: Student Enrollment and Teacher Statistics Forecasting Based on Time-Series Analysis
Source: Comput Intell Neurosci. 2020 Sep 15;2020:1246920. doi: 10.1155/2020/1246920 (PMC7520033; doi:10.1155/2020/1246920)
Supplement: Supplementary Materials — The supplementary figures demonstrate (1) the number of students enrolled (Supplementary Figure 1) and full-time equivalent teachers hired (Supplementary Figure 2) in Taiwanese public and private educational institutions; (2) student-teacher ratio in public (Supplementary Figure 3) and private (Supplementary Figure 4) educational institutions; and (3) the trend of student and teacher statistics in public and private educational institutions based on level (Supplementary Figures 5–8). [file 1246920.f1.docx]

**Student Enrollment and Teacher Statistics Forecasting based on Time Series Analysis**


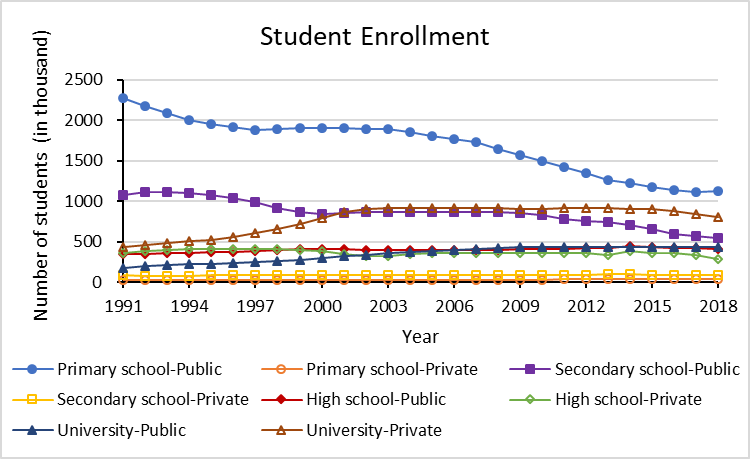


Fig. 1 Student Enrollment in Taiwan


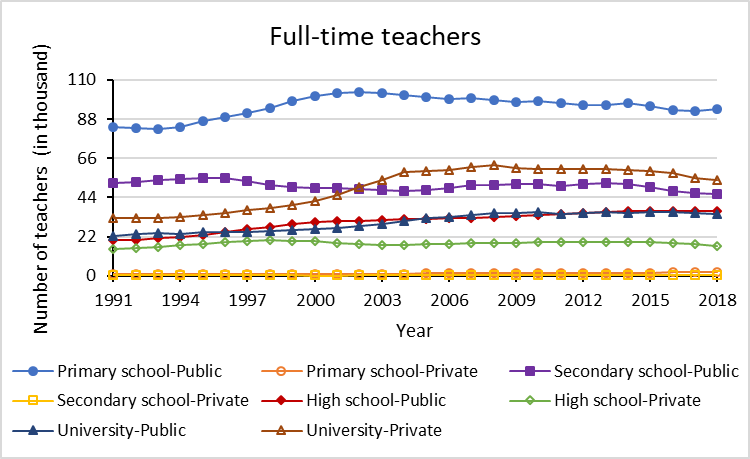


Fig. 2 Full-time teachers in Taiwan


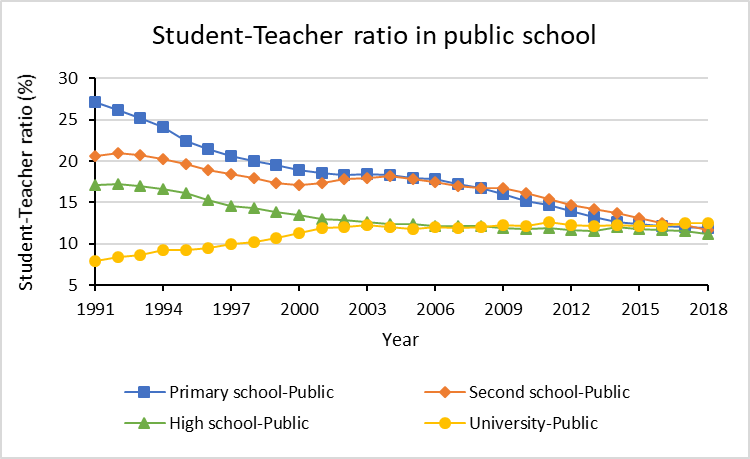


Fig. 3 Annual Student-Teacher ratio in Taiwan school (Public)


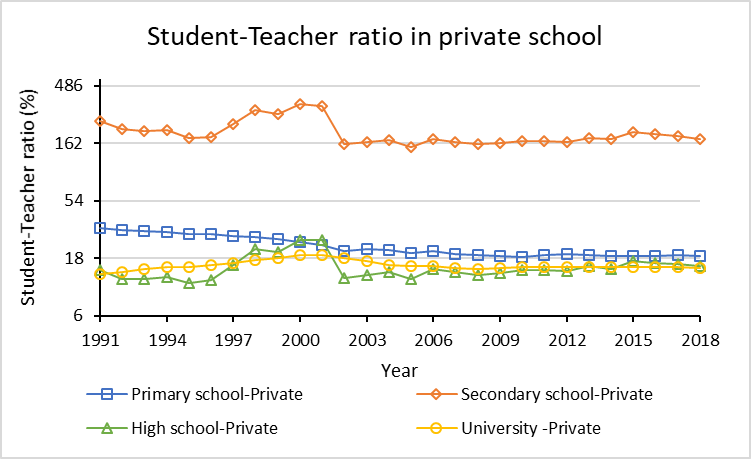


Fig. 4 Annual Student-Teacher ratio in Taiwan school (Private)


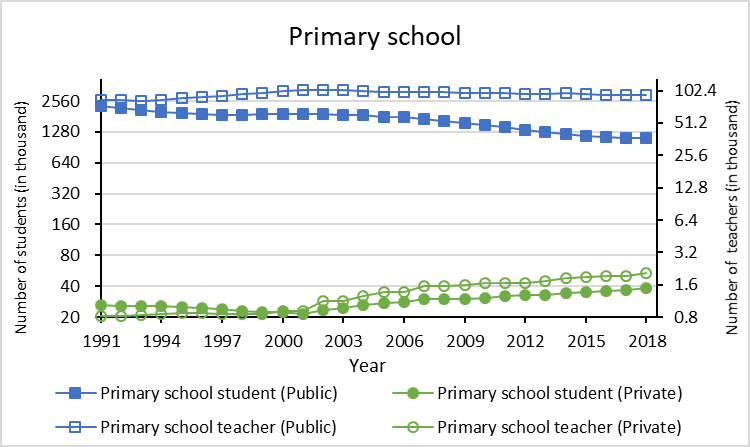


Fig. 5 Number of student and teacher (public school and private school)


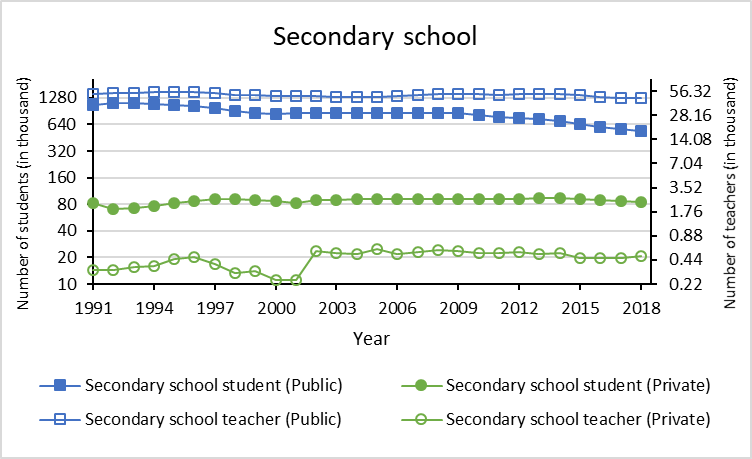


Fig. 6 Number of student and teacher (public school and private school)


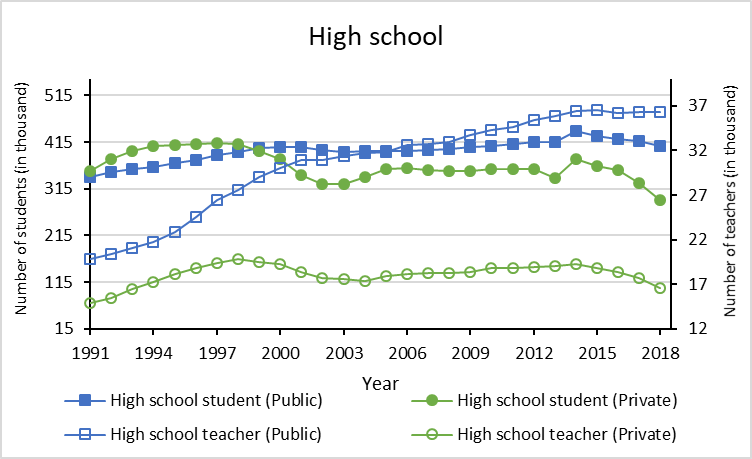


Fig. 7 Number of student and teacher (public school and private school)


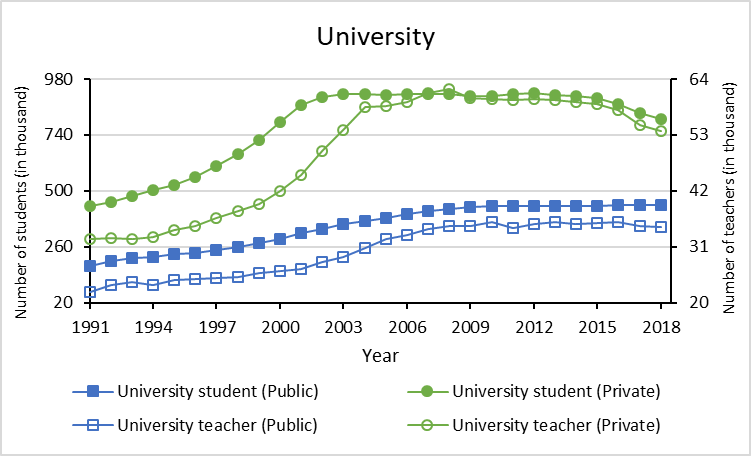


Fig. 8 Number of student and teacher (public school and private school)
